# Flavor properties of Chinese noodles processed by dielectric drying

**DOI:** 10.3389/fnut.2022.1007997

**Published:** 2022-09-29

**Authors:** Qian Lin, Aiqing Ren, Rui Liu, Yanan Xing, Xiuzhu Yu, Hao Jiang

**Affiliations:** ^1^College of Food Science and Engineering, Northwest A&F University, Yangling, China; ^2^Institute of Food Research, Hezhou University, Guangxi, China; ^3^Cereal Industrial Technology Academy, Hebei Jinshahe Flour and Noodle Group/Hebei Cereal Food Processing Technology Innovation Centre, Xingtai, China; ^4^Engineering Research Center of Grain and Oil Functionalized Processing, Universities of Shaanxi Province, Yangling, China

**Keywords:** Chinese dried noodles, volatile compounds, E-nose, HS-GC-MS, HS-GC-IMS

## Abstract

Volatile organic compounds (VOCs) significantly impact food flavor. In this work, Electron nose (E-nose), head space solid phase microextraction-gas chromatography-mass spectrometry (HS-SPME-GC-MS), and head space-gas chromatography-ion mobility spectrometry (HS-GC-IMS) techniques were applied to analyze different drying effects: microwave, hot air, and radio frequency on the aroma of Chinese noodles. E-nose analysis suggests that aromatic differences are mainly from broad range-methane. HS-SPME-GC-MS and HS-GC-IMS identified 47 and 26 VOCs in the fresh and dried noodles, respectively. The VOCs in the dried noodles were mainly aldehydes, alcohols, and esters. Drying significantly reduced the types of VOCs in Chinese dried noodles. Microwave dried noodles exhibited the strongest aroma after the shortest time of treatment, suggesting microwave drying may be the best drying method for noodles. Using aromatic analysis, this paper provides useful information for understanding the flavor of flour products and offers new ideas for drying noodles.

## Introduction

Wheat is one of the three most widely consumed crops in the world. Asian countries make about 12% of the world’s wheat into noodles (1). As a traditional staple food, Chinese dried noodles are widely eaten in many Asian countries because of their easy preservation, convenient consumption, and high nutritional value. Existing studies have analyzed an impressive array of factors associated with Chinese dried noodles: physical and chemical properties; quality improvement; evaluation methods; and changes in storage (2). However, the aromatic characteristics of Chinese dry noodles and the effects of these changes on product quality characteristics are rarely discussed.

Drying is a key step in Chinese dried noodles manufacturing, prolonging the shelf-life of foods. In addition, the volume and weight of dehydrated food reduce the cost of processing, packaging, and transporting. There are some disadvantages to the hot air (HA) drying process, such as slow heat transfer, long running time, high energy consumption, reduced nutrition, and mitigated taste, among other things (3). The current research directions for Chinese dried noodles are mainly to optimize the drying parameters (4) and change the noodle recipe to improve the quality and enrich the flavor of Chinese dried noodles. To reduce the loss of flavor during drying and to overcome the disadvantages of HA drying, it has become necessary to research new drying methods in addition to the two directions mentioned above.

Microwave (MW, 2450 MHz) drying—a subset of dielectric dehydration technology—uses electromagnetic radiation for rapid heating and drying (5). Dielectric heating absorbs electromagnetic waves and converts them into heat energy, a process that greatly improves heating efficiency. Carvalho et al. (6) reduced the drying time of HA drying malt by 95 percent using MW drying. MW drying can not only save energy but also better retain the aromatic components of food materials (7). Pongpichaiudom and Songsermpong (8) compared the quality of the noodles in terms of cooking quality, textural characteristics, and color. The results showed that MW dried noodles have good attributes, including minimal cooking losses. However, there is a paucity of research on the flavor of MW dried noodles. MW drying does have some inherent shortcomings, including uneven heating and limited penetration depth. These issues are a result of the uneven distribution of the electromagnetic field in the heating bin and the uneven distribution of water in the target material. Some methods have been suggested to overcome these drawbacks. Shen et al. (9) proposed a method that combined ventilation convection with MW heating to achieve an even distribution of temperature and water content, thus improving the uniformity of drying.

A low frequency electromagnetic drying called radio frequency (RF, 27 MHz) is an alternative to MW that addresses some of these more pressing issues (10). Compared with MW drying, RF drying has many advantages, such as good heating uniformity, significant penetration depth because of its longer wavelengths, stable product temperature control, and high cost-effectiveness. Jiang et al. (11) dried strawberries using RF to prove the advantages of RF drying on drying rate and nutrient retention. Wang et al. (12) also proved the potential of RF technology in the drying uniformity and quality of agricultural products by drying nuts. During these experiments, RF was less efficient than MW because of the lower frequency. Compared to HA drying, RF exhibited a poorer drying uniformity because of overheating in corners, edges, and centers, although RF performed far better than MW in terms of uniformity (13). Because RF drying performs far better than MW in terms of uniformity and other aspects, RF is now applied to many aspects of food preparation. Zhang et al. (14) demonstrated that RF heated meat batters were significantly harder, chewier, and gummier. Zhang et al. (15) testified that RF drying gives food a new texture, such as crispy and chewy. However, there is a large gap in the application of RF drying to noodles, especially the effect of dielectric on the flavor of dried noodles has rarely been reported.

Aroma is one of the most important indicators of food quality, while volatile organic compounds (VOCs) are the most important factors impacting flavor (16). To date, two analytical methods (sensory analysis and instrumental analysis) have been used to analyze VOCs in food products. In contrast to subjective sensory analysis, instrumental analysis can be used to explore VOCs at the molecular level. The electronic nose (E-nose) is an aroma detection system that provides fast sensory information on food. E-nose allows efficient, cost-effective, and non-destructive identification of food products. It has been used in a wide range of applications such as freshness and spoilage assessment, classification, and adulteration identification of food products (17, 18). Headspace solid-phase microextraction-gas chromatography-mass spectrometry (HS-SPME-GC-MS) is a technique that combines enrichment extraction with separation and detection for the qualitative and quantitative detection of VOCs in food products (19). It has been widely used in food analysis, such as traceability analysis and identification of species and VOCs in food and oil processing (20). headspace-gas chromatography-ion mobility spectrometry (HS-GC-IMS) is of interest because it combines the high sensitivity of gas chromatography with the fast response time of ion mobility spectrometry. HS-GC-IMS has been applied to the monitoring of food processing and the assessment of aroma changes during food storage (21). Yet limited research exists on the effects of different drying methods on the flavor of Chinese dried noodles. To improve the understanding, this study analyzed different drying techniques—including HA, MW, and RF—on noodles and the flavor thereof. E-nose, HS-SPME-GC-MS, and HS-GC-IMS were used to detect VOCs in Chinese dried noodles.

## Materials and methods

### Samples

Flour was provided by the Hebei Jinshahe flour industry group. The flour had a moisture content of 13.04% ± 0.12 (w.b.) and protein content of 12.78%. The edible salt used in noodles comes from the local supermarket (Yangling, Shaanxi). The phenethyl acetate was purchased from Sigma-Aldrich (73747, St. Louis, MO, USA). All chemical reagents used in this study are analytical grade.

### Preparation of fresh noodles

Two hundred gram flour, 1% salt, and 30% water are mixed using a dough mixer (KMM760, Kenwood, London, UK) for 4 min. After mixing, the dough was calendered on the experimental noodle machine (BJM-6, Deqing Baijie Electric Appliance Co., Ltd, Huzhou, Zhejiang, China). The calendering process includes several steps: 1.5 mm axial spacing calendering three times, including direct calendering once, and folded calendering twice. The materials were then placed in a self-sealing bag and allowed to incubate for 30 min. After incubation, noodles were passed through the noodle machine for four times to get noodles with a width of 2 mm and a thickness of 1 mm.

### Drying treatment

#### Microwave drying

Twenty-five gram of fresh noodles samples were hung on the noodles rack and placed in a commercial microwave oven (MM823ESJ-SA, Midea Group, Guangdong, China). Samples were dried at 500 W power for 4 min with a heating intensity of 20 W/g. After removal from the oven, samples were placed in the dryer and immediately cooled to room temperature.

#### Hot air drying

Hundred gram of fresh noodles samples were hung on the drying rack and placed in the drying oven (GZX-9023MBE, Shanghai Boxun Industrial Co., LTD. Medical Equipment Factory, Shanghai, China). Referring to the method of Zhang et al. (4) and optimization, the drying process was split into three stages: pre-drying, main drying, and final drying. Pre-drying lasts for 40 min at 25°C; the main drying takes 140 min at 45°C, and the final drying lasts 60 min at 30°C.

#### Radio frequency drying

Three hundred grams of fresh samples were placed in a plastic container (300 × 220 × 60 mm). This container sat in the center of the RF equipment, which was set to 27.12 MHz and 6 KW (SO6B, Streffield International, Wokingham, UK). The distance between the plates was 110 mm. The heating lasted 120 min, and the heating intensity was 20 W/g. During the heating process, the six-channel optical fiber temperature sensor system (HQ-FTS-D120, Xi’an Haier Technology Co., Ltd., Xi’an, China) was used to measure the sample temperature with an accuracy of ± 0.5 °C. The optical fiber sensor was set at the four corners of the sample surface and the core of the sample.

### Determination of moisture content

During the drying process, the drying curve is measured by weight analysis. The weight was recorded after the sample was removed from the drying chamber. Moisture loss was assessed at 30 s, 30, and 20 min intervals during MW, HA, and RF drying respectively. The weight change was recorded until the weight difference between the two measurements was less than 0.1 g. Consider this as the termination point. After each drying experiment, the dried noodles were heated at 105°C in a dryer until complete desiccation to calibrate the moisture content, ensuring the accuracy of the experimental data.

The moisture content (1) and drying rate (2) of the sample is calculated as follows:


(1) $$\mathrm{Moisture}\,\mathrm{content}=\left(M_{t}-M_{d}\right)/M_{d}$$

(2) $$D\,r\,y\,i\,n\,g\,r\,a\,t\,e=\frac{M_{t+d\,t}-M_{t}}{d\,t}$$

M_*t*_ and M_*t*__+__*dt*_ was the mass of material (g) at drying time t and t + dt, respectively; and M_*d*_ was the absolute dry mass of the material (g).

### Electron nose analysis

The E-nose analysis was carried out using E-nose equipment with a sensor array system (Airsense Analytics GmbH., Schwerin, Germany). The system is composed of ten metal oxide semiconductors with different chemical compositions and thicknesses. These were used to determine the flavor of the Chinese dried noodles treated by different drying methods. Weighed 3 g sample into a 20 mL headspace bottle. After equilibration at 25°C for 24 h, an electronic nose probe was inserted and the air at the top is sampled. The headspace of the bottle was gradually absorbed and replaced by clean air. The volatile gas was transmitted to the detector at a constant rate for 60 s until the sensor signal reached a stable value. Cleaned the detector with clean air between each sample for 300 s, or until the sensor signal returned to baseline. Each sample was measured at least 10 times.

### Headspace solid phase microextraction-gas chromatography-mass spectrometry

According to the method described by Zhao et al. (17) the HS-GC-MS instrument was used for sample analysis, but with a slight modification. The Chinese dried noodles (2 g) were weighed and put into a 20 mL headspace bottle. Then, added 15 μL of phenyl ethyl acetate as an internal standard (final concentration 4.09 × 10^4^ μg/L). Sealed the bottle and allowed an incubation period of 15 min. Afterward, a solid-phase micro-extraction sampler with a carbox-en/divinylbenzene/polydimethylsiloxane (CAR/DVB/PDMS) fiber (Supelco Ltd., Pennsylvania, PA, USA) was used to extract the VOCs in the headspace for 20 min at 40°C. The sampler was inserted into the gas chromatograph injector and thermally desorbed at 250°C for 3 min in split free injection mode. Identification and quantitative analysis were performed using the Gas chromatography-Mass Spectrometer Ultra system (QP2010, Shimadzu, Kyoto, Japan). Used the DB-1 MX capillary column (60 m × 0.25 mm × 0.25 μm) for separation. The injection port temperature was 250°C and the injection occurred in non-shunt mode. Helium (≥99.999%) was used as a carrier gas at a constant flow rate of 1 mL/min. The programmed temperature was set to 40°C for 3 min. Following this period, the temperature increased by 4°C/min to 120°C. Then, the temperature grew by 6°C/min to 240°C, where it held constant for 12 min. The mass spectrum conditions sustained an interface temperature of 280°C. The ion source temperature was 230°C. The full scanning range was m/z 50–500. The names and related information for different volatile chemical components were determined by searching the mass spectrometry computer data system and matching the standard mass spectrometry database. The retention indices of each initially characterized substance were used and compared to the retention indices of the corresponding substances reported in the literature for further characterization. The expected concentration of each chemical component was obtained by the internal standard method.

### Headspace-gas chromatography-ion mobility spectrometry

Based on Guo et al. (22) the Agilent 490 Gas chromatograph (Agilent Technologies, Palo Alto, CA, USA) and IMS instrument (FlavourSpec^®^, Gesellschaft für Analytische Sensorsysteme mbH, Dortmund, Germany) were used for sample analysis, but with slight modification. Noodles (2.0 g) were placed in a 20 mL headspace glass sampling bottle. Then added 15 μL phenylethyl acetate as an internal standard (final concentration 4.09 × 10^4^ μg/L). Subsequently, these samples were incubated at 60°C for 15 min. After incubation, the automatic injection device (CTC Analytics AG, Zwingen, Switzerland) injected 500 μL of top air into the syringe (85°C, no shunt mode). Then, the sample was driven into the MTX-5 capillary column (15 m × 0.53 mm) with a nitrogen flow (≥99.999%) under isothermal conditions of 60°C. IMS instrument was used to complete the analysis. The % samples followed a standard progression: 2 mL/min for 2 min; 10 mL/min for 8 min; 100 mL/min for 10 min; 100 mL/min for 5 min. The flow of bleaching gas (nitrogen) in the bleaching pipe was 150 mL/min. All analyses were repeated 3 times. VOCs were identified by comparing RI and the standard drift time (the time in milliseconds taken for ions to reach the collector through the drift tube) in the GC-IMS library.

### Data analysis

The obtained data were analyzed by Excel (version 2016, Microsoft, Redmond, WA, USA), and the charts were drawn by Origin software (version 2019b, Microcal Inc., Massachusetts, MA, USA). The E-nose data were processed by E-nose software (Winmuster 1.6.2.5, Airsense Analytics GmbH, Schwerin, Germany). GC-MS and GC-IMS used the built-in software database to determine the names and relevant information of different volatile chemical components.

## Results and discussion

### Moisture content

**FIGURE 1:**
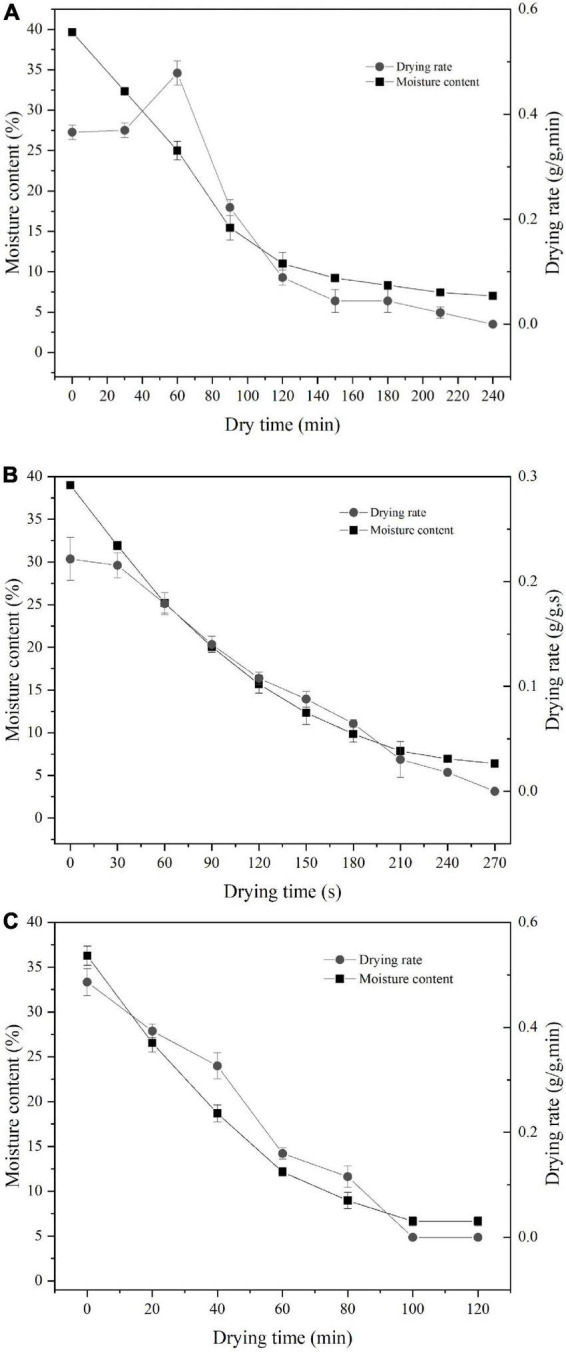
Effects of different drying methods on noodle drying curve and drying rate curve. **(A)** HA, **(B)** MW, and **(C)** RF.

Results showed that the total drying times of MW, HA, and RF were 4, 240, and 120 min, respectively (Figure 1). HA drying could be divided into three stages: pre-drying, main, and final. Pre-drying evaporates part of the water on the surface of wet noodles in order to fix the tissues and avoid the noodles’ deformation. The main drying stage had the highest drying temperature and removed moisture from the noodles quickly. This stage allowed for the rapid removal of moisture from the surface of the noodles. If the drying rate is too rapid, the surface of the wet noodles will shrink, rendering the noodles dry outside and wet inside. The main function of the final drying stage was to balance the differences in moisture and temperature inside and outside of the noodles and to eliminate the various internal stresses caused by the drying and shrinking of the noodles.

The drying rate of dielectric drying methods (MW and RF) was significantly higher than that of HA drying. This was due to different energy transfer modes. For HA drying, external heat flux was applied to the product surface, resulting in slow heat penetration from the product surface to the interior. MW and RF drying caused the internal and surface temperature of the material to rise rapidly and synchronously (23). Therefore, the drying rate of MW and RF was significantly higher than that of HA drying. The frequency of MW was higher than that of RF, which means MW exhibits a greater heating rate. Among all drying methods under the same heating intensity, MW drying had the highest drying rate.

The moisture content of Chinese dried noodles in all drying methods gradually decreased as the drying process. The lack of a constant drying period in all methods was probably due to the low thickness of the Chinese dried noodles not providing a constant amount of moisture, indicating that the drying process is mainly controlled by the diffusion of moisture. With advanced and efficient drying technology, MW and RF, especially MW, could shorten the drying time by 98% compared to HA.

### E-nose analysis

**FIGURE 2:**
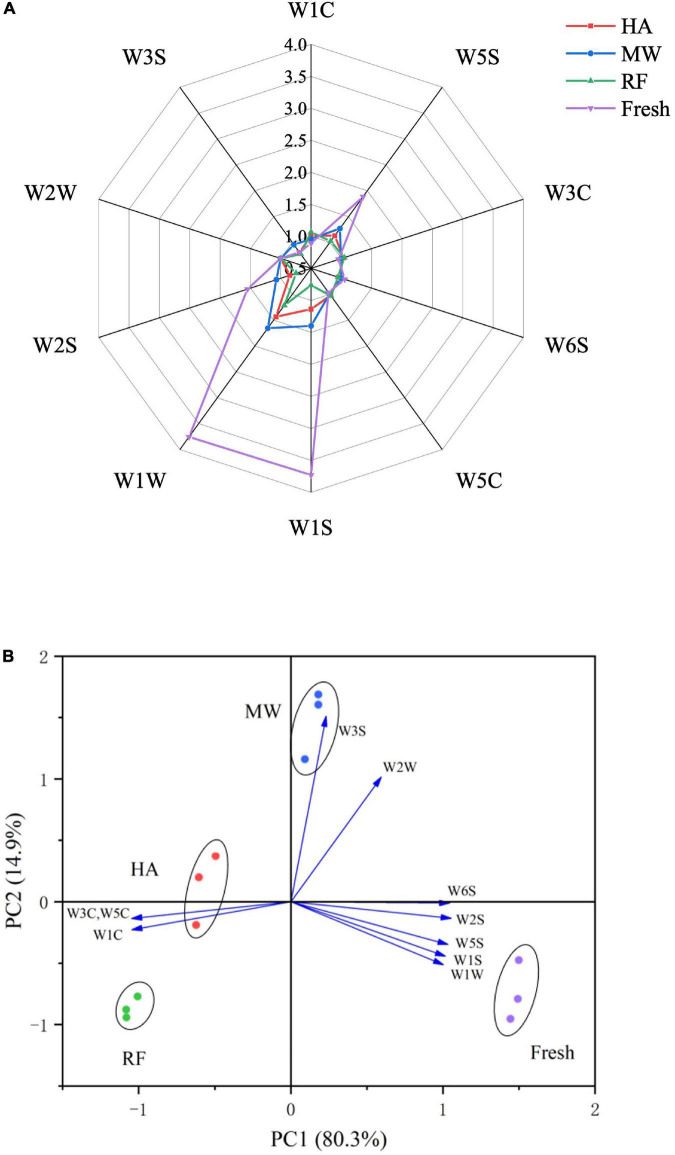
E-nose analysis. **(A)** Radar map of fresh and dried noodles. **(B)** Principal component analysis of fresh noodles and dry noodles.

E-nose analysis reported the real-time response value of each sensor, which changes gradually with time. The responses of 10 sensors to these samples showed different characteristics. According to Figure 2, drying changed the content and types of VOCs in fresh noodles. Most of the sensors for fresh noodles seem to display stronger signals than they do for dried noodles, indicating that the smell of fresh noodles is the most complex. For dried noodles, W1W (mainly sensitive to terpene compounds) and W1S (broad range-methane) had the highest response values. Comparing noodles dried by different methods, the signal difference between W1W and W1S sensors was the strongest. This suggested that the differences in basic noodle flavor mainly come from inorganic sulfides, alcohols, aldehydes, and ketones. During the drying process, the Maillard reaction between sugars, proteins, and amino acids continues, resulting in a large number of heterocyclic compounds, such as furans. These compounds enhanced the signal of W1C (aromatic ring, benzene) and affected the flavor of noodles, giving dry noodles a burnt flavor and sweetness.

In order to evaluate the difference of VOCs in each sample, the E-nose response data set was analyzed by principal component analysis (PCA) (Figure 2B). The spatial regions of these samples showed that the flavor of fresh and dried noodles varied in significant ways. The cumulative contribution rate of the first two principal components accounted for 95.2% of the total variance, indicating that these alone were sufficient to reflect the original multi-index information. Principal factor 1 (PC1) explained 80.3% of the variance in the data, and principal factor 2 (PC2) explained 14.9% of the variance. HA and RF dried noodles data were located in the negative region of PC1. According to the Figure 2B, W3C (mainly sensitive to aromatic ammonia), W5C (mainly sensitive to an alkane, aromatics, and small polar compounds), and W1C sensors were distributed in the negative region of PC1, which indicates that aromatic compounds contributed more to HA dried noodles and RF dried noodles than other types of VOCs. Fresh noodles and MW dried noodles were located in the positive area of PC1. W5S (mainly sensitive to nitrous oxides), W6S (mainly sensitive to hydrocarbons), W5C (mainly sensitive to an alkane, aromatics, and small polar compounds), W1S, W1W, W2S (mainly sensitive to most alcohols, aldehydes, and ketones), W2W (mainly sensitive to aromatics and organic sulfides) and W3S (mainly sensitive to long-chain alkanes) sensors were distributed in the positive area of PC1. Fresh noodles and MW dried samples contained more sulfur compounds, broad-spectrum methyl compounds (aldehydes, ketones), and broad-spectrum alcohols, indicating that MW has the best flavor retention. This finding was consistent with the results of radar map generation. The E-nose response results highlighted the great flavor differences between samples that experienced different drying methods. But this method was limited in its ability to identify specific compounds that affect characteristic flavor. Volatiles and aromatic active compounds needed to be further studied to determine the impact of each drying method on key aromatic compounds—which ultimately result in flavor variations.

### Chromatography-mass spectrometry analysis

The composition and content of the VOCs formed under different drying regimes were measured using HS-SPME-GC-MS. According to the retention index, retention time, and mass spectral data of standard compounds and the MS library, a total of 47 VOCs were identified in fresh noodles and noodles dried by three methods (Table 1). These VOCs could be divided into seven categories: 11 kinds of aldehydes, 8 kinds of alcohols, 7 kinds of ketones, 4 kinds of esters, 10 kinds of hydrocarbons, 6 kinds of acids, and 1 kind of furan. The main composition of wheat noodles was consistent with a previous study on the VOCs analysis of noodles (24).

**TABLE 1:** Comparisons of the detected VOCs in fresh and Chinese dried noodles by HS-SPME-GC-MS.

No	Compounds name	CAS	Formula	Molecular weight	RI	Estimated concentration (μg/kg fresh noodles)			
						Fresh	MW	HA	RF
	**Aldehydes**								
1	Acetaldehyde	75-07-0	C_2_H_4_O	44.05	408	7.49	–	–	–
2	Paraldehyde	513-86-0	C_6_H_12_O_3_	132.16	717	14.89	–	–	–
3	Hexanal	66-25-1	C_6_H_12_O	100.16	806	79.14	1579.21	1230.81	247.12
4	Pentanal	110-62-3	C_5_H_10_O	86.13	707	3.83	169.70	–	–
5	Heptaldehyde	111-71-7	C_7_H_14_O	114.19	905	–	–	–	10.27
6	1-Nonanal	124-19-6	C_9_H_18_O	142.24	1104	–	329.56	263.93	107.19
7	(*2E*)-2-Nonenal	18829-56-6	C_9_H_16_O	140.22	1112	6.55	–	196.99	102.28
8	4-Octadecenal	56554-98-4	C_18_H_34_O	266.46	1766	–	45.21	–	–
9	Decanal	112-31-2	C_10_H_20_O	156.27	1204	9.13	263.29	149.63	80.10
10	Pentadecanal	2765-11-9	C_15_H_30_O	226.40	1701	5.37	–	–	8.86
11	(*Z*)-Hexadec-9-enal	56219-04-6	C_16_H_30_O	238.41	1808	–	–	–	7.37
	**Alcohols**								
12	Ethanol	64-17-5	C_2_H_6_O	46.07	463	29.25	439.94	439.35	312.43
13	1-Penten-3-ol	616-25-1	C_5_H_10_O	86.13	671	16.91	–	–	–
14	1-Pentanol	71-41-0	C_5_H_12_O	88.15	761	154.31	411.35	69.46	-
15	1-Hexanol	111-27-3	C_6_H_14_O	102.17	860	380.64	200.72	–	–
16	2-Hydroxycineole	18679-48-6	C_10_H_18_O_2_	170.25	1247	8.92	84.03	38.51	12.80
17	1-Octen-3-ol	3391-86-4	C_8_H_16_O	128.21	969	45.54	–	–	–
18	3,5-Octadien-2-ol	69668-82-2	C_8_H_14_O	126.2	1385	42.85	–	–	–
19	Phenethy-l-alcohol	60-12-8	C_8_H_10_O	122.16	1934	9.18	–	–	–
	**Ketones**								
20	5-Methyl-2-hexanone	110-12-3	C_7_H_14_O	114.19	789	–	171.65	80.60	53.74
21	2-Heptanone	110-43-0	C_7_H_14_O	114.19	853	27.76	–	–	–
22	3-Methyl-3-buten-2-one	814-78-8	C_5_H_8_O	84.12	797	25.34	–	–	–
23	2-Methyl-4-heptanon	626-33-5	C_8_H_16_O	128.21	888	–	454.70	113.58	–
24	2-Octanone	111-13-7	C_8_H_16_O	128.21	952	5.14	–	–	–
25	Geranylacetone	3796-70-1	C_13_H_22_O	194.31	1420	–	88.71	18.04	30.30
26	3,5-Octadiene-2-one	38284-27-4	C_8_H_12_O	124.18	968	6.81	–	–	–
	Esters								
27	2-Amino-propionic acid ethyl ester	17344-99-9	C_5_H_11_NO_2_	117.15	1097	282.15	416.62	148.56	60.13
28	Isobutyl formate	542-55-2	C_5_H_10_O_2_	102.13	718	–	1491.38	–	–
29	(*E*)-2-Hexenyl hexanoate	53398-86-0	C_12_H_22_O_2_	198.30	1389	7.66	–	–	–
30	Dibutyl phthalate	84-74-2	C_16_H_22_O_4_	278.34	2037	13.54	175.44	–	24.41
	**Hydrocarbons**								
31	Pentane	109-66-0	C_5_H_12_	72.15	518	479.35	5319.61	–	–
32	2-Methylpentane	107-83-5	C_6_H_14_	86.18	554	5.25	115.05	–	9.25
33	3-Methylpentane	96-14-0	C_6_H_14_	86.18	554	37.79	52.11	35.64	6.59
34	Hexane	110-54-3	C_6_H_14_	86.18	618	–	–	431.55	125.22
35	Heneicosane	629-94-7	C_21_H_44_	296.57	2109	11.39	63.47	–	–
36	Eicosane	112-95-8	C_20_H_42_	282.55	2008	–	–	11.96	6.25
37	Hentriacontanone	630-04-6	C_31_H_64_	436.84	3103	7.11	–	9.72	–
38	Dodecane	112-40-3	C_12_H_26_	170.33	1214	9.23	70.24	–	–
39	Tetradecane	629-59-4	C_14_H_30_	198.39	1413	7.81	61.95	46.77	–
40	Dipentene	5989-27-5	C_10_H_16_	136.23	1018	14.40	60.69	21.92	–
	**Acids**								
41	Hexanoic acid	142-62-1	C_6_H_12_O_2_	116.16	974	34.99	72.94	49.29	24.36
42	Acetic acid	64-19-7	C_2_H_4_O_2_	60.05	576	–	–	102.01	17.00
43	Octanoic acid	124-07-2	C_8_H_16_O_2_	144.21	1173	–	46.50	–	5.16
44	Nonanoic acid	112-05-0	C_9_H_18_O_2_	158.24	1272	–	41.94	14.59	5.89
45	Decanoic acid	112-37-8	C_10_H_20_O_2_	172.26	1471	–	–	19.51	–
46	Tridecanlic acid	638-53-9	C_13_H_26_O_2_	214.34	1681	–	–	–	9.90
	**Furan**								
47	2-Pentylfuran	3777-69-3	C_9_H_14_O	138.21	1040	10.18	81.12	21.99	11.93

MW, microwave dried noodles; HA, hot air dried noodles; RF, radio frequency dried noodles.

**FIGURE 3:**
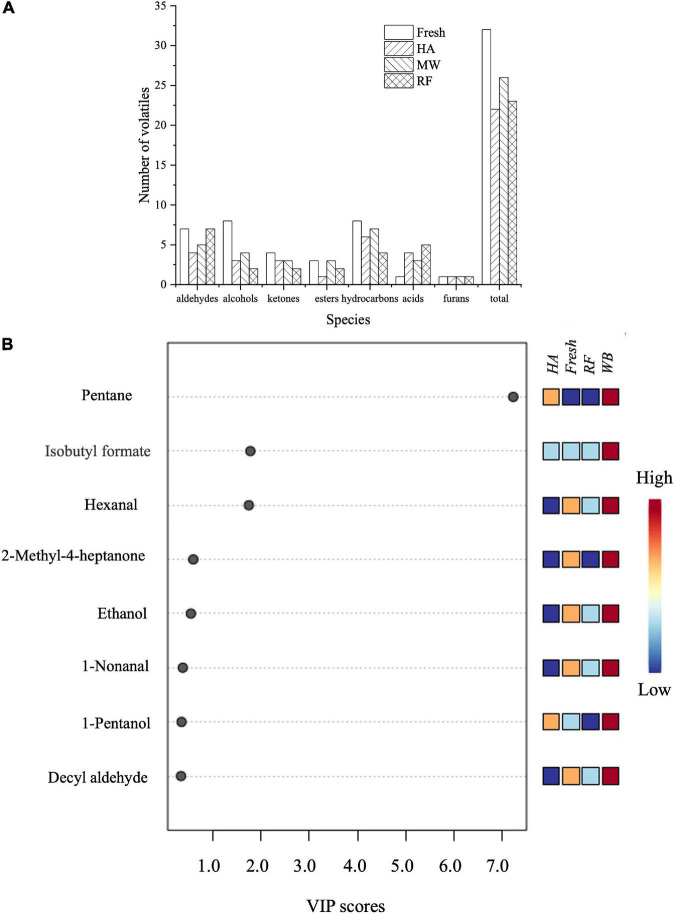
Comparison of VOCs in fresh and dried noodles by HS-SPME-GC-MS. **(A)** Numbers of VOC types. **(B)** VIP scores in PLS-DA.

The quantity of VOCs in fresh noodles differs from VOCs in dried noodles (Figure 3). There were 32 kinds of VOCs in fresh noodles (including three esters, seven aldehydes, eight alcohols, four ketones, eight hydrocarbons, one acid, and one furan). There were 22 VOCs in HA dried noodles, including one ester, four aldehydes, three alcohols, three ketones, six hydrocarbons, four acids, and one furan. MW dried noodles contained 26 VOCs, including three esters, five aldehydes, four alcohols, three ketones, seven hydrocarbons, three acids, and one furan. RF dried noodles contained 23 kinds of VOCs, including two esters, seven aldehydes, two alcohols, two ketones, four hydrocarbons, five acids, and one furan. Data showed that the type of drying treatment significantly altered the types of VOCs in noodles. Acids increased after HA, MW, and RF drying. The variety of furan did not change after drying but their concentration increased. All other substances showed a decreasing trend in species after drying. In addition, some substances disappeared completely after drying by different methods (e.g., acetaldehyde and paraldehyde). This suggests that drying can effectively eliminate the grassy odor present in fresh noodles. Nonanal, moreover, can only be detected after drying. Nonanal added a pleasant floral and fruity fragrance to the noodles and made the dried noodles more flavorful.

Using the PLS-DA, which provided a value for VIP, a total of 8 differential marker flavor substances were selected from 60 volatile substances. These substances might be what caused the significant flavor difference between differently dried noodles. As shown in Figure 3B, among these eight VOCs, there were three aldehydes (hexanal, 1-nonanal, and decyl aldehyde), one ester (isobutyl formate), two hydrocarbons (2-methyl-4-heptanon, and pentane), and two alcohols (ethanol, and 1-pentanol). Consistent with the results of Zhang et al. (25), changes in aldehydes during drying had a great impact on flavor. The lipoxygenase oxidation of unsaturated fatty acids by lipoxygenase was a major pathway for the formation of aldehydes (26). Minimal increase in aldehydes after RF drying of fresh noodles. Probably because the maximum temperature of RF drying was 90°C and lipoxygenase was inactivated at 80 and 90°C for holding times above 3 min (27), thus inhibiting the production of aldehydes.

Esters also contributed to a large extent to the noodles. The content of ester compounds produced during MW drying was higher than the content produced in HA and RF drying. It is noteworthy that isobutyl formate, which produces a fruity flavor, was detected in the MW dried noodles and was not detected in any of the other noodle samples. Generally, esters were one of the degradation products of unsaturated fatty acids that occur late in the oxidation process (28). Most of the esters formed by short chain acids provided a fruity flavor. Esters formed by long chain acids offered a light greasy flavor. Unlike MW, HA drying significantly reduced the content of esters detected, and no new esters were formed. In addition, alcohol compounds revealed an inverse trend with drying. Drying significantly reduced the type and content of alcohol substances. These substances played an auxiliary role in the overall flavor composition of Chinese dried noodles. Compared with other kinds of compounds, hydrocarbon compounds did not alter significantly between fresh and dried noodles. Hydrocarbon compounds provide a limited contribution to the flavor quality of the noodles.

Changes in flavor substances were caused by lipid degradation and oxidation, Maillard reactions, and enzymatic reactions (29). Aldehydes, alcohols, and esters were the main VOCs that create noodle flavor differences. A comparison of flavor substances across the drying methods revealed that the aroma of MW dried noodles was the strongest. Different drying methods had different water loss rates. In addition, drying process time and temperature affect flavor variation. High temperature inhibited the oxidative decomposition of unsaturated fatty acids and affected the formation of aldehydes (30). The MW method took only 4 min, less than 2% of the HA drying time. This was relevant as long exposure to heat treatment adversely impacts noodle flavor.

### Chromatography-ion mobility spectrometry analysis

#### Identification of volatile organic compounds by chromatography-ion mobility spectrometry

**FIGURE 4:**
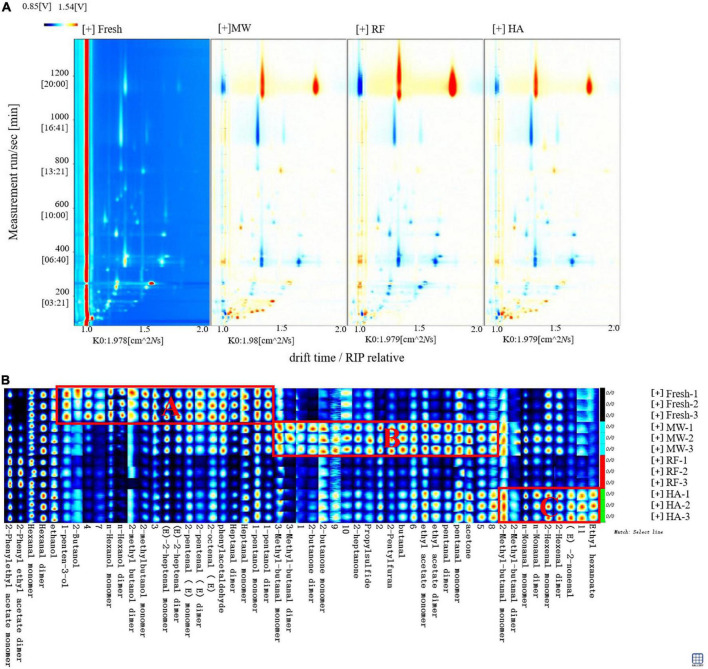
Comparison of VOCs in fresh and dried noodles by GC-IMS. **(A)** Topographic plots of Noodle drying. **(B)** The fingerprint of volatile organic compounds in fresh and dried noodles.

Volatile organic compounds in fresh and dried noodles were analyzed by HS-GC-IMS. HS-GC-IMS and HS-SPME-GC-MS exhibited different VOCs identification capabilities. Chen et al. (31) proved that HS-SPME-GC-MS was more sensitive to pyrazines, while HS-GC-IMS measured aldehydes and ketones more accurately. Using topographic map derivation, created a difference map to identify VOCs changes. Each point area showed a different retention time (*y*-axis), relative drift time (*x*-axis), and signal strength. The redder the area, the greater the signal strength. The bluer the area, the smaller the signal strength (Figure 4). Based on the National Institute of Standards and Technology library, VOCs were identified by comparing the corresponding retention index and drift time.

After the noodles dry, most of the red signals appeared in the retention time range below 200 s, and the drift time is 1.0–1.5. The results showed that a large number of VOCs were formed during drying. Considering that signal separation was mainly related to polarity—and the retention time of polar compounds is concentrated in the range of 100–200 s—this result indicated that drying may contribute to the formation of polar compounds. In addition, RF dried noodles samples displayed a blue signal in the 100–400 s, which was significantly higher than MW drying and HA drying. RF samples displayed a red signal lower than that of MW and HA samples. This showed that RF drying had the worst flavor retention of the three drying methods, a finding which is consistent with the results of GC-MS.

Head space-gas chromatography-ion mobility spectrometry detected 51 areas, 40 of which were identified; the other 11 areas were not confirmed. It was worth noting that 14 chemicals existed in the form of monomers and dimers [heptanal, 1-pentanol, 2-butanone, 2-methyl-butanal, 2-methylbutanol, (*E*)-2-pentenal, (*E*)-2-heptenal, 2-hexenal, 3-methyl-butanal, ethyl acetate, hexanal, *n*-hexanol, *n*-nonanal and pentanal]. Twenty-six kinds of VOCs were identified in both fresh and dried noodles (Table 2). These included 6 alcohols, 13 aldehydes, 3 ketones, 2 esters, and 1 compound containing sulfur. It should be noted that the content of VOCs in dimer form is often lower than that of monomer form, indicating that the stability of the dimer form is inferior to that of monomer form. It could be that the dissociation transition of dimer monomer was more likely to occur than the degradation of monomer during the drying process (32).

**TABLE 2:** Head space-gas chromatography-ion mobility spectrometry integration parameters of volatile compounds in fresh and Chinese dried noodles.

No.	Compound	CAS	Formula	Molecular weight	RI	Rt [sec]	Dt [RIP rel]	Peak volume (a. u.)			
								Fresh	MW	HA	RF
	**Aldehydes**										
1	Butanal	123-72-8	C_4_H_8_O	72.10	590.40	133.81	1.29	154.21	635.13	388.12	116.25
2	3-Methyl-butanal monomer	590-86-3	C_5_H_10_O	86.10	645.40	157.84	1.17	1195.75	1754.21	938.56	682.47
3	3-Methyl-butanal dimer	590-86-3	C_5_H_10_O	86.10	646.00	158.11	1.40	147.94	528.09	114.22	58.42
4	2-Methyl-butanal monomer	96-17-3	C_5_H_10_O	86.10	668.30	167.83	1.18	415.22	627.82	637.94	496.59
5	2-Methyl-butanal dimer	96-17-3	C_5_H_10_O	86.10	666.40	167.02	1.38	76.85	211.79	182.63	84.66
6	Pentanal monomer	110-62-3	C_5_H_10_O	86.10	691.40	178.90	1.19	1366.84	1838.61	1686.40	1143.18
7	Pentanal dimer	110-62-3	C_5_H_10_O	86.10	690.00	177.82	1.42	680.70	1878.67	1186.09	294.06
8	(*E*)-2-Pentenal monomer	1576-87-0	C_5_H_8_O	84.10	744.10	221.02	1.10	492.27	426.12	338.76	215.81
9	(*E*)-2-pentenal dimer	1576-87-0	C_5_H_8_O	84.10	743.50	220.50	1.36	113.01	62.45	49.66	19.62
10	Hexanal monomer	66-25-1	C_6_H_12_O	100.20	789.00	258.25	1.26	3307.85	3120.62	3029.02	3152.21
11	Hexanal dimer	66-25-1	C_6_H_12_O	100.20	789.00	258.25	1.56	7939.69	9223.81	9274.95	5084.26
12	2-Hexenal monomer	505-57-7	C_6_H_10_O	98.10	845.00	320.01	1.18	628.45	623.39	696.85	333.35
13	2-Hexenal dimer	505-57-7	C_6_H_10_O	98.10	843.10	317.85	1.51	128.01	130.11	168.72	51.51
14	Heptanal monomer	111-71-7	C_7_H_14_O	114.20	898.90	383.95	1.34	2336.14	1726.92	1539.99	902.74
15	Heptanal dimer	111-71-7	C_7_H_14_O	114.20	897.10	380.70	1.68	1349.68	764.49	614.27	175.68
16	(*E*)-2-Heptenal monomer	18829-55-5	C_7_H_12_O	112.20	954.50	482.69	1.25	2762.68	2754.50	1903.04	1457.92
17	(*E*)-2-Heptenal dimer	18829-55-5	C_7_H_12_O	112.20	953.10	480.05	1.66	1169.65	980.76	502.38	234.51
18	*n*-Nonanal monomer	124-19-6	C_9_H_18_O	142.20	1105.10	768.70	1.48	1370.32	2848.27	3365.48	2498.12
19	*n*-Nonanal dimer	124-19-6	C_9_H_18_O	142.20	1105.40	769.36	1.94	103.90	328.02	464.48	247.60
20	Phenylacetaldehyde	122-78-1	C_8_H_8_O	120.20	1039.00	639.90	1.26	431.94	438.08	230.48	205.82
21	(*E*)-2-octenal	2548-87-0	C_8_H_14_O	126.20	1064.70	690.10	1.33	492.29	390.20	380.40	264.10
22	(*E*) -2-nonenal	18829-56-6	C_9_H_16_O	140.20	1161.00	877.60	1.40	239.30	347.66	642.96	268.71
	**Alcohols**										
23	Ethanol	64-17-5	C_2_H_6_O	46.10	513.10	100.06	1.05	4890.46	4400.35	5114.11	4273.30
24	2-Butanol	78-92-2	C_4_H_10_O	74.10	590.70	133.94	1.15	340.68	129.59	129.96	90.07
25	1-Penten-3-ol	616-25-1	C_5_H_10_O	86.10	685.00	175.12	0.94	719.18	411.18	375.57	283.77
26	2-Methylbutanol monomer	137-32-6	C_5_H_12_O	88.10	733.30	212.38	1.24	1345.37	536.44	604.16	303.66
27	2-Methyl butanol dimer	137-32-6	C_5_H_12_O	88.10	732.30	211.57	1.48	234.51	109.16	132.78	115.12
28	1-Pentanol monomer	71-41-0	C_5_H_12_O	88.10	769.10	240.91	1.25	2664.65	2232.20	2174.30	1471.84
29	1-Pentanol dimer	71-41-0	C_5_H_12_O	88.10	767.70	239.82	1.51	1942.41	1299.55	1044.89	418.53
30	*n*-Hexanol monomer	111-27-3	C_6_H_14_O	102.20	873.90	351.80	1.33	5152.66	3858.82	2780.85	2074.24
31	*n*-Hexanol dimer	111-27-3	C_6_H_14_O	102.20	877.80	356.14	1.64	3317.66	1421.44	902.39	396.66
	**Ketones**										
32	Acetone	67-64-1	C_3_H_6_O	58.10	519.30	102.76	1.12	855.15	1631.75	1879.06	699.04
33	2-Butanone monomer	78-93-3	C_4_H_8_O	72.10	578.00	128.41	1.07	583.94	1101.97	820.49	634.48
34	2-Butanone dimer	78-93-3	C_4_H_8_O	72.10	582.30	130.30	1.24	91.71	579.76	244.76	87.60
35	2-Heptanone	110-43-0	C_7_H_14_O	114.20	889.00	368.42	1.26	260.18	747.61	506.66	245.34
	**Esters**										
36	Ethyl acetate monomer	141-78-6	C_4_H_8_O_2_	88.10	597.20	136.78	1.10	696.06	1132.11	1108.32	744.64
37	Ethyl acetate dimer	141-78-6	C_4_H_8_O_2_	88.10	596.60	136.51	1.33	204.00	634.49	583.87	132.23
38	Ethyl hexanoate	123-66-0	C_8_H_16_O_2_	144.20	999.00	561.96	1.34	187.27	136.78	334.07	133.00
	**Furan**										
39	2-Pentylfuran	3777-69-3	C_9_H_14_O	138.20	991.00	547.42	1.25	122.66	294.23	158.84	95.68
	**Ether**										
40	Propylsulfide	111-47-7	C_6_H_14_S	118.20	895.30	377.43	1.15	135.72	206.52	151.46	96.99

RI, the retention index; Rt, the retention time; Dt, the drift time; MW, microwave dried noodles; HA, hot air dried noodles; RF, radio frequency dried noodles.

#### Comparison of volatile organic compounds fingerprints of samples

The complete VOCs of the fresh and dried noodles in three different ways could be obtained by fingerprint and VOCs differences could be compared intuitively and quantitatively. It could be seen from Figure 4B that the main VOCs flavor substance of fresh noodles are concentrated in region A. These VOCs were used to distinguish the flavor differences between samples. Main substances included five alcohols (1-penten-3-ol, 2-butanol, n-hexanol, 2-methylbutanol and 1-pentanol) and five aldehydes ((*E*)-2-heptenal, (*E*)-2-pentena, (*E*)-2-octenal, phenylacetaldehyde and heptanal). Shahidi et al. (33) pointed out that most alcohols, except ethanol, were the result of lipid oxidation. Unsaturated alcohol was one of the main flavor substances in cereal products, which had been detected in dried noodles. However, alcohol compounds had less effect on the taste of noodles than aldehydes and had a synergistic effect on the overall taste of noodles. The main formation pathway of aldehydes was the oxidative decomposition of unsaturated fatty acids (e.g., oleic acid, linoleic acid, and linolenic acid) by hydroperoxide isomerase and lipoxygenase. Some aldehydes were also produced by the oxidative degradation of esters. The detection concentration of aldehydes was high, which will produce a pleasant smell, such as grass and fruit. Similar to the results of Li et al. (34), aldehydes contributed greatly to noodle flavor, more so than other compounds do; aldehydes were likely the main flavor contributor in noodles.

Different drying methods highlighted differences in alcohols, aldehydes, and ketones. Similar VOCs were found in MW and HA dried samples. The substances in area C can represent VOCs that were characteristic of HA dried noodles. These substances included four aldehydes [2-methylbutyraldehyde, nonanal, hexanal, and (*E*)-2-nonanal] and one ester (ethyl caproate). Compared with fresh noodles, HA dried noodles possessed a less significant amount of alcohol, which was lost due to vaporization during the drying process (35). The structure of enols was generally unstable and easy to isomerize into stable carbonyl compounds (36), which changed the types of aldehydes present during HA drying. Aldehydes had high reactivity, which was one of the reasons for the volatile flavor during drying. During HA drying, short chain lower aldehydes are transformed into higher fatty aldehydes above C8. These fatty aldehydes have a stronger aroma of oil, nuts, grass, and fruit (37). In dried noodles, certain kinds of aldehydes increased significantly, which was consistent with the results of GC-MS. Esters are an important component of VOCs and are the main carrier of odor (38). Ester compounds are mainly formed through the esterification of alcohols and fatty acids. HA drying produced ethyl caproate, which added a strong aroma of liquor and pineapple to the noodles (39).

Of the three drying methods, MW retained the most amounts of VOCs present in fresh noodles. The substances in area B were used to represent the characteristic VOCs of MW dried noodles. These substances included three aldehydes (3-methylbutyraldehyde, butyraldehyde, and glutaraldehyde), three ketones (2-butanone, 2-heptanone, and acetone), one ether (propylene sulfide), one heterocyclic compound (2-pentylfuran), and one ester (ethyl acetate). Noodles aroma was informed largely by aldehydes (40). Compared with fresh noodles, MW noodles contained fewer types of alcohol and aldehyde, but, in general, the types of VOCs in MW dried noodles were more abundant than in fresh noodles. Ketones, ethers, and heterocyclic compounds were the characteristic substances of MW dried noodles. The main formation pathways of ketones are the oxidation of fatty acids, the oxidation of alcohols, and the decomposition of esters (41). The ketone compounds detected in MW dried noodles mostly contributed to a fruit flavor. Heterocyclic compounds are a series of products, intermediates, and derivatives of the Maillard reaction, caramelization reaction, and Strecker degradation reaction. Some intermediates of the Maillard reaction further degrade with lipids to form a series of derived compounds, such as 2-*n*-pentyl furan. Heterocyclic compounds create a roasting aroma (42). They were the main compounds that form the wheat aroma of wheat products. Even in small amounts, sulfur compounds often produce very strong aromas; propyl sulfide was one of the main flavor contributors to MW dried noodles, adding an ether flavor.

Radio frequency dried noodles had fewer kinds and lower concentrations of VOCs, suggesting that RF drying VOCs retention was inferior to MW and HA drying. The fruit flavor ketones (acetone, 2-butanone) detected in MW dried noodles were also produced by RF drying, but the threshold of the ketones was high and their flavor contribution was limited. Wang et al. (43) found that RF dried strawberries better retained color and nutrients (e.g., carotenoids, anthocyanins, and total phenols), but poorly retained flavor, a finding which matches our own. Most fresh noodles’ characteristic VOCs were decreased or went undetected following RF drying. RF drying also changed short chain lower aldehydes into long chain higher fatty aldehydes [(*E*)-2-nonanal and nonanal], but to a far less than HA drying. During RF drying, when the local temperature of the sample exceeded 80°C, the activities of enzymes such as lipoxygenase decreased significantly (44), a state which is not conducive to aldehyde formation. This may contribute to the poor flavor retention of RF. In addition, in the RF drying process, noodles samples were exposed to high temperatures for an extended period. Exposure time impacted the degree of thermal damage during food heating and drying. Although the high temperature is conducive to the Maillard reaction, it also promotes the volatilization of substances with a boiling point of 50–80°C (e.g., acetone and 2-pentylfuran). This contributed to the low detection of flavor substances in RF noodles. Dielectric heating results in rapid energy coupling with food moisture and leads to fast heating and drying. Hemis et al. (45) studied the effect of MW drying on wheat drying kinetics. The results showed that product quality increases with the decrease in drying time. Therefore, inferred that the poor retention of flavor substances by RF drying compared with MW drying may be due to the significant increase in drying time. With these factors in mind, MW drying may be the best drying method to optimize noodle flavor.

## Conclusion

The results showed that the drying efficiency of dielectric drying methods (MW and RF) significantly exceeded that of HA drying. The E-nose, HS-SPME-GC-MS, and HS-GC-IMS technologies were used to qualitatively and quantitatively analyze fresh and dried noodles (MW, RF, and HA), and the effects of these drying methods on the flavor of dried noodles were determined and discussed. The results of E-nose showed that the basic flavor differences of noodles mainly came from inorganic sulfides, alcohols, aldehydes, and ketones. HS-SPME-GC-MS and HS-GC-IMS identified 47 and 26 VOCs in various noodle samples, respectively. The results showed that fresh noodles and noodles dried by HA, MW, and RF had different aroma characteristics, and the three types of drying methods had a significant effect on the aroma characteristics of noodles. MW drying best retained these critical flavor substances, while RF drying resulted in lower concentrations of VOCs. Considering flavor retention, drying efficiency, and production capacity, MW drying is the appropriate drying method. There remains a lack of research on the effects of different drying methods on noodle flavor. The results provide a useful basis for the aroma quality analysis of noodles. The effect of MW and RF on the texture and structure of noodles needs to be further investigated.

## Data availability statement

The original contributions presented in this study are included in the article/supplementary material, further inquiries can be directed to the corresponding author.

## Author contributions

HJ developed the idea of the work. HJ and QL designed the study and drafted the original manuscript. AR and RL searched the literature. XY and HJ critically revised and improved the manuscript. All authors contributed to the article and approved the submitted version.
